# Introduction of Avian Influenza A(H6N5) Virus into Asia from North America by Wild Birds

**DOI:** 10.3201/eid2511.190604

**Published:** 2019-11

**Authors:** Sol Jeong, Dong-Hun Lee, Yu-Jin Kim, Sun-Hak Lee, Andrew Y. Cho, Jin-Yong Noh, Erdene-Ochir Tseren-Ochir, Jei-Hyun Jeong, Chang-Seon Song

**Affiliations:** University of Connecticut, Storrs, Connecticut, USA (S. Jeong, D.-H. Lee);; Konkuk University, Seoul, South Korea (S. Jeong, Y.-J. Kim, S.-H. Lee, A.Y. Cho, J.-Y. Noh, E.-O. Tseren-Ochir, J.-H. Jeong, C.-S. Song)

**Keywords:** avian influenza, intercontinental transmission, wild bird, phylogenetic analysis, influenza viruses, Asia, North America, wild birds, influenza, viruses, South Korea

## Abstract

An avian influenza A(H6N5) virus with all 8 segments of North American origin was isolated from wild bird feces in South Korea. Phylogenetic analysis suggests that this virus may have been introduced into Asia by wild birds, highlighting the role of wild birds in the dispersal of these viruses.

Avian influenza viruses (AIVs) have evolved into phylogenetically independent lineages as a result of separated distribution and migration of wild waterfowl, which are the natural reservoir of the viruses, because of natural geographic barriers. However, the ecologic separation of the migrating hosts seems incomplete, as some species of wild waterfowl (e.g., Northern pintails) migrate across the Bering Strait and serve as an intercontinental bridge for AIV between Eurasia and North America (*1*). Evidence for intercontinental exchange of AIV has been reported more frequently along continental margins where migratory flyways overlap, such as western Alaska (*2*); previous studies have described the exchange of gene segments or dispersal of complete virus between the 2 continents through migratory bird movements (*2*–*4*). We report detection of a fully North American AIV A(H6N5) subtype from South Korea during 2017. 

During September 2017–March 2018, a total of 4,403 fresh fecal samples were collected in wild bird habitats of South Korea as part of an annual surveillance program for AIV. The sampling was focused on waterfowl at freshwater habitats such as rivers, streams, and reservoirs. We screened the samples for influenza A virus by real-time reverse transcription PCR (rRT-PCR) targeting the matrix gene and by embryonated chicken egg inoculation. A total of 131 samples tested positive for the matrix gene rRT-PCR; 58 AIV subtypes were isolated, including 1 H1N1, 6 H1N2, 2 H1N3, 1 H3N8, 4 H5N2, 3 H5N3, 40 highly pathogenic H5N6 (*5*), and 1 H6N5. We sequenced full-length genomes of the isolates using next-generation sequencing, as described previously (*5*). We deposited the nucleotide sequences of A/Mandarin duck/Korea/K17-1638-5/2017(H6N5) virus (hereafter 1638-5/2017 virus) in GenBank (accession nos. MK830100–7). 

The Gimpo area (37°43′37″N, 126°39′54″E), from where the H6N5 virus was isolated, is one of the major wintering sites of such migratory birds as the greater white-fronted goose (*Anser albifrons*), bean goose (*Anser fabalis*), mallard (*Anas platyrhynchos*), and Eastern spot-billed duck (*Anas poecilorhyncha*) (Appendix 1 Figure 1). We identified the host of influenza A virus–positive feces using a DNA barcoding technique (*5*). We conducted comparative phylogenetic analysis of the 1638-5/2017 virus to trace its origin. For each segment, we aligned sequences with most of the closest full-length related sequences obtained from BLAST (https://blast.ncbi.nlm.nih.gov/Blast.cgi) and used these sequences for phylogenetic analysis. We constructed maximum-likelihood phylogenetic trees using the general time-reversible plus gamma substitution model with 1,000 bootstrap replications in RAxML version 8 (*6*).

Search results in the BLAST database indicated that all 8 viral gene segments of the 1638-5/2017 virus shared >98% nucleotide identity with North American wild bird AIV collected during 2015–2017 (Appendix 1 Table). Only genetic sequences of North American ancestry virus were in the sequencing data from 1638-5/2017 virus, suggesting the absence of contamination of viral genes from Eurasian-lineage virus during the sampling and virus isolation. Consistent with these findings, maximum-likelihood phylogenetic analysis indicated that all 8 gene segments clustered together with the sequences of AIV isolated from North American wild birds during 2016–2017, rather than those of Eurasian isolates (Figure; Appendix 1 Figure 2; Appendix 2). These results strongly suggest the introduction of AIV of North American ancestry into Eurasia by wild birds.

**Figure:**
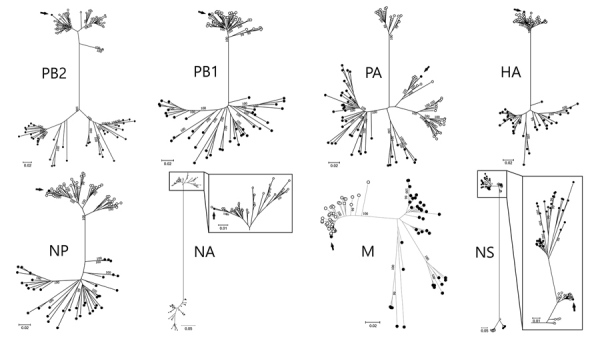
Maximum-likelihood phylogenetic trees indicating relationships between Eurasian (black circles) and North American (white circles) lineages of avian influenza A viruses. Black arrows indicate genome sequences of A/Mandarin duck/Korea/K17-1638–5/2017(H6N5) virus isolated in this study. Bootstrap values >99% are shown. Maximum-likelihood phylogenetic trees with complete strain names are shown in Appendix 1 Figure 2. Scale bar indicates nucleotide substitutions per site. HA, hemagglutinin gene; M, matrix gene; NA, neuraminidase gene; NP, nucleoprotein gene; NS, nonstructural gene; PA, polymerase acidic gene; PB, polymerase basic gene.

The high nucleotide similarity of each gene segment with North American AIV identified during 2015–2017 suggests that the genome constellation of the 1638-5/2017 virus had been recently dispersed from North America to Eurasia. Mandarin ducks (*Aix galericulata*) are found mainly in the Far East and Southeast Asia, within the East Asian–Australasian flyway, and are not recognized as making regular movements between Eurasia and North America. The 1638-5/2017 virus might have been introduced from North America into Eurasia by wild birds migrating between Eurasia and North America and then been transmitted to Mandarin ducks.

Repeated reports of intercontinental transmission of AIV, particularly detections of fully Eurasian AIV, including H8N4 (*4*) and H9N2 (*7*); H16N3 (*8*) and highly pathogenic H5Nx (*3*) in North America; and the fully North American H6N5 virus in South Korea (this study) suggest that dispersal of AIV between Eurasia and North America is bidirectional and might not be exceedingly rare (*2*,*7*). Bahl et al. reported that the multiple introductions of Eurasian H6 AIV resulted in the establishment of viral sublineages in the North American AIV gene pool and changed the evolutionary dynamics of AIV in wild birds in North America (*9*). In addition, the North American N8 subtype gene has been established in wild bird populations migrating through the East Asian–Australasian flyway and was identified from a chicken farm and live bird market in South Korea (*10*). Enhanced influenza surveillance along migratory flyways and complete genome sequencing of identified viruses would be essential for better understanding of the intercontinental migrations of AIV and for early detection of the introduction of novel strains.

Appendix 1 Additional genetic information about wild birds infected with avian influenza A(H6N5) virus.

Appendix 2 List of influenza virus isolates used in this study from the GISAID database.
