# Supplementary material for: Introduction of Avian Influenza A(H6N5) Virus into Asia from North America by Wild Birds
Source: Emerg Infect Dis. 2019 Nov;25(11):2138–40. doi: 10.3201/eid2511.190604 (PMC6810209; doi:10.3201/eid2511.190604)
Supplement: Appendix 1 — Additional genetic information about wild birds infected with avian influenza A(H6N5) virus. [file 19-0604-Techapp-s1.pdf]

Article DOI: https://doi.org/10.3201/eid2512.190604

Introduction of Avian Influenza A(H6N5) Virus into Asia from North America by Wild Birds

Appendix

Appendix Table. Isolates sharing the highest nucleotide identity with each segment of A/Mandarin duck/Korea/K17–1638–5/2017(H6N5) found in GenBank database on April 15, 2019.

Gene*	Strain name	GenBank accession no.	nt identity
PB2	A/glaucous-winged gull/Southcentral Alaska/ 16MB03648/2016(H5N2)	CY239407.1	99%
PB1	A/American black duck/Maryland/16OS2661/ 2017(H7N3)	MK236794.1	99%
PA	A/mallard/Alberta/71/2017(H3N8)	MH411975.1	99%
HA	A/northern pintail/California/HS052B/2015(H6N5)	KY983161.1	99%
NP	A/American green-winged teal/Missouri/ 17OS3212/2017(H3N8)	MK236738.1	99%
NA	A/American wigeon/California/HS010/2015 (H12N5)	KY983225.1	98%
M	A/mallard/Alberta/390/2017(H1N1)	MH637401.1	99%
NS	A/northern pintail/Alaska/16–041335–6/2016(H5N2)	MH546895.1	99%

*HA, hemagglutinin; M, matrix; NA, neuraminidase; NP, nucleoprotein; NS, nonstructural; PA, polymerase acidic; PB, polymerase basic

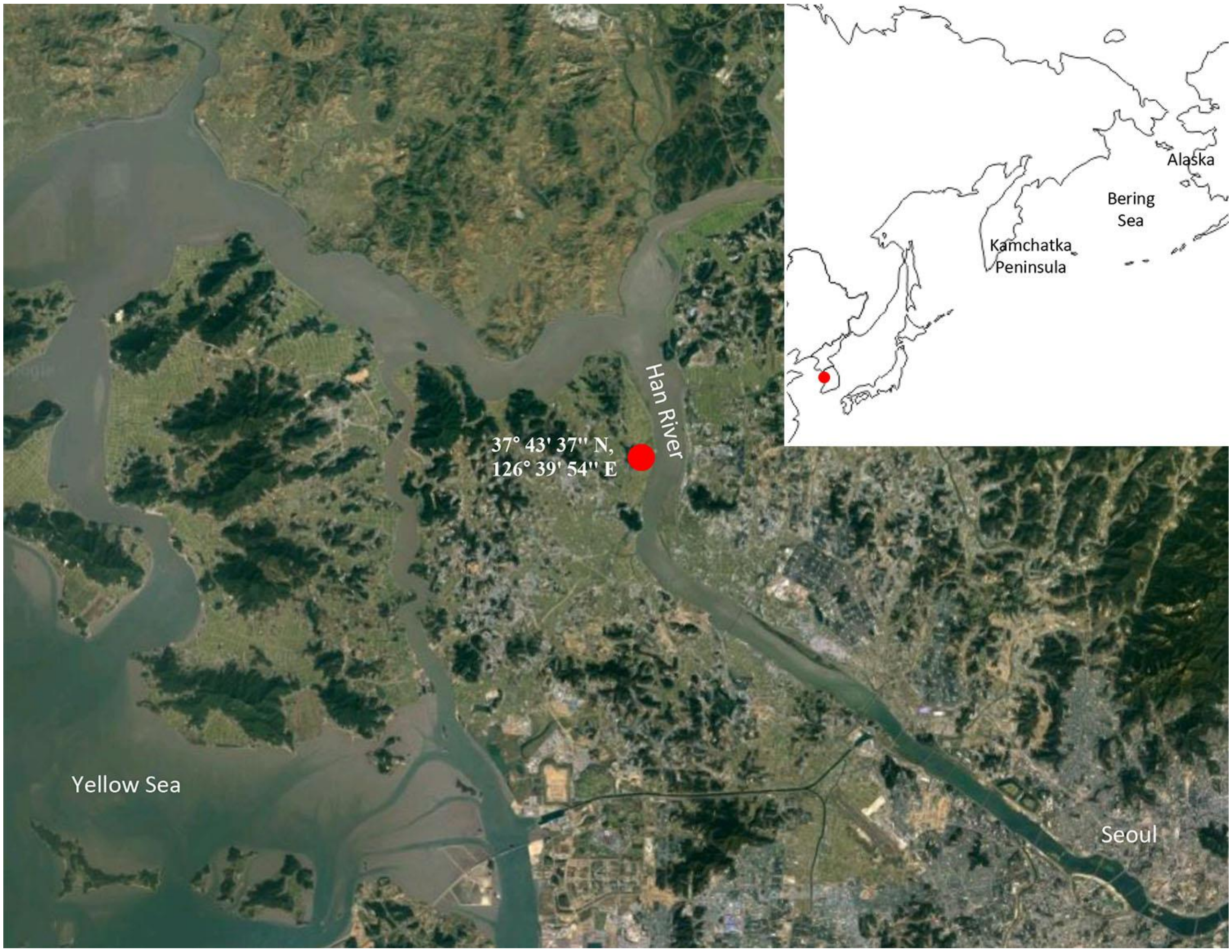

Appendix Figure 1. Sampling location of A/Mandarin duck/Korea/K17–1638–5/2017(H6N5) virus (indicated with red circle) in South Korea. Map was retrieved from Google Maps (2019 June 28), https://www.google.com/maps/@37.7003952,126.6354144,74246m/data=!3m1!1e3!5m1!1e4?hl=en.

A

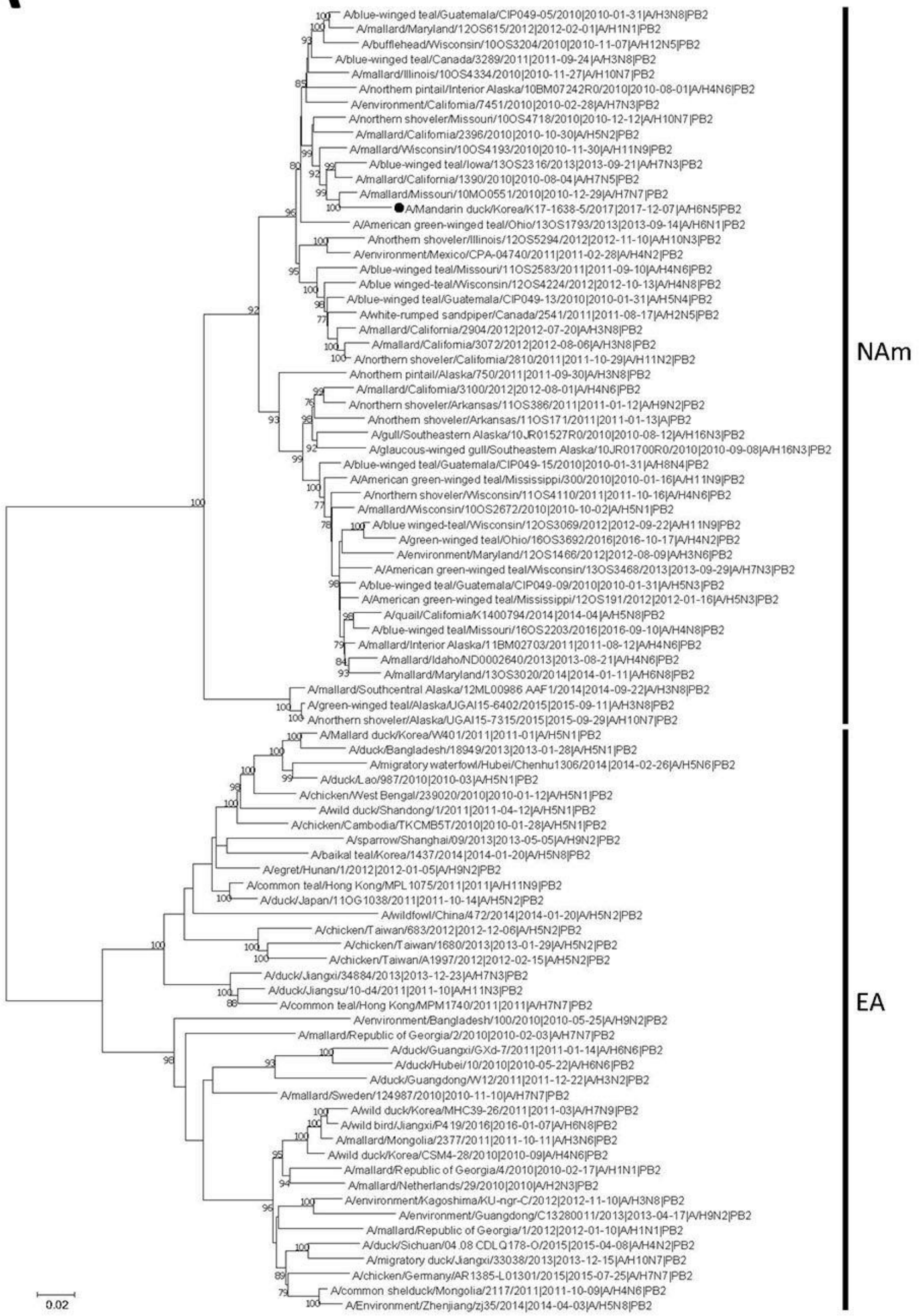

B

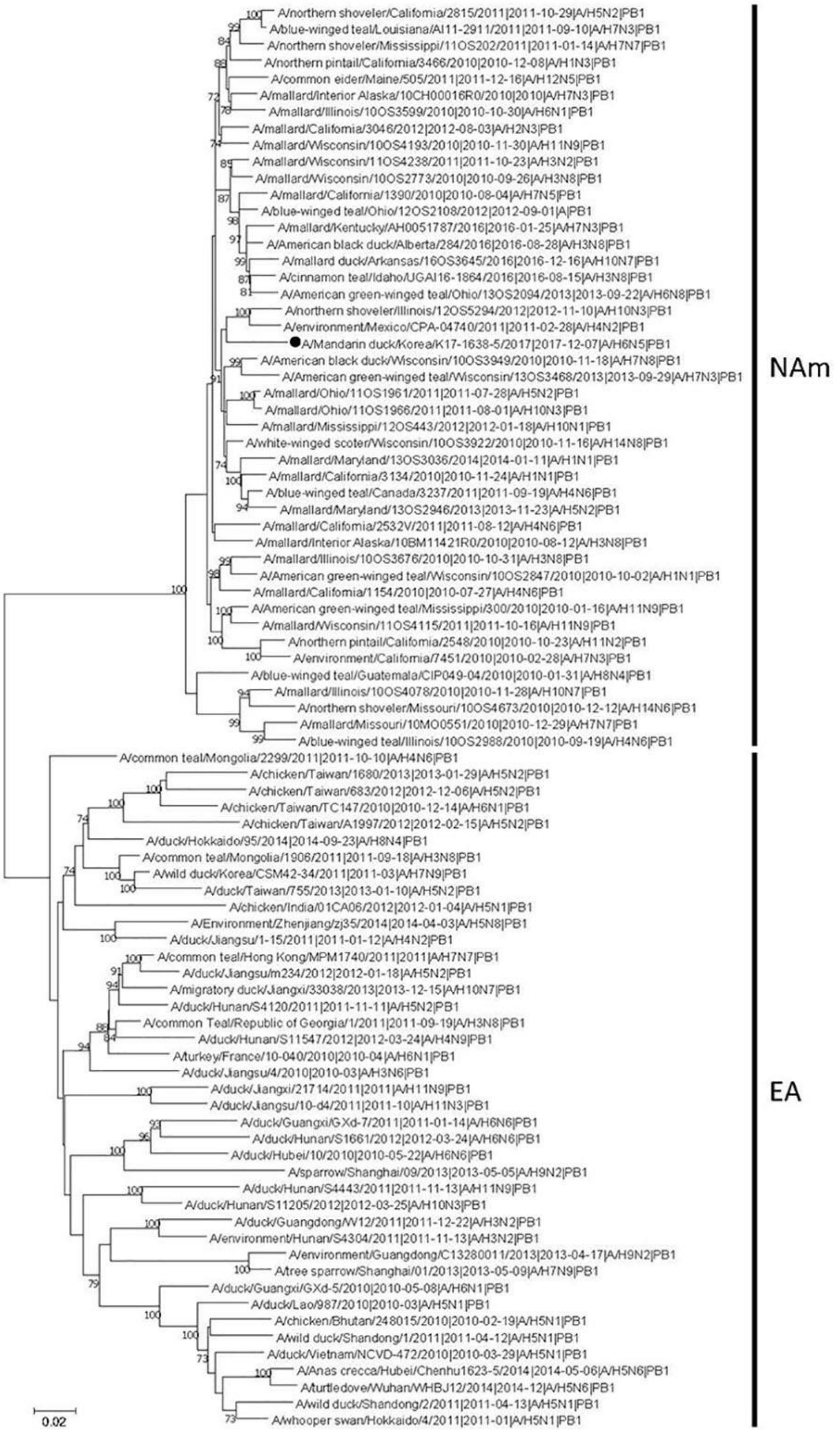
A/northern shoveler/California/2815/2011|2011-10-29|A/H5N2|PB1
A/blue-winged teal/Louisiana/AI11-2911/2011|2011-09-10|A/H7N3|PB1
A/northern shoveler/Mississippi/11OS202/2011|2011-01-14|A/H7N7|PB1
A/northern pintail/California/3466/2010|2010-12-08|A/H1N3|PB1
A/common eider/Maine/505/2011|2011-12-16|A/H12N5|PB1
A/mallard/Interior Alaska/10CH00016R0/2010|2010|A/H7N3|PB1
A/mallard/Illinois/10OS3599/2010|2010-10-30|A/H6N1|PB1
A/mallard/California/3046/2012|2012-08-03|A/H2N3|PB1
A/mallard/Wisconsin/10OS4193/2010|2010-11-30|A/H11N9|PB1
A/mallard/Wisconsin/11OS4238/2011|2011-10-23|A/H3N2|PB1
A/mallard/Wisconsin/10OS2773/2010|2010-09-26|A/H3N8|PB1
A/mallard/California/1390/2010|2010-08-04|A/H7N5|PB1
A/blue-winged teal/Ohio/12OS2108/2012|2012-09-01|A|PB1
A/mallard/Kentucky/AH0051787/2016|2016-01-25|A/H7N3|PB1
A/American black duck/Alberta/284/2016|2016-08-28|A/H3N8|PB1
A/mallard duck/Arkansas/16OS3645/2016|2016-12-16|A/H10N7|PB1
A/cinnamon teal/Idaho/UGAI16-1864/2016|2016-08-15|A/H3N8|PB1
A/American green-winged teal/Ohio/13OS2094/2013|2013-09-22|A/H6N8|PB1
A/northern shoveler/Illinois/12OS5294/2012|2012-11-10|A/H10N3|PB1
A/environment/Mexico/CPA-04740/2011|2011-02-28|A/H4N2|PB1
A/Mandarin duck/Korea/K17-1638-5/2017|2017-12-07|A/H6N5|PB1
A/American black duck/Wisconsin/10OS3949/2010|2010-11-18|A/H7N8|PB1
A/American green-winged teal/Wisconsin/13OS3468/2013|2013-09-29|A/H7N3|PB1
A/mallard/Ohio/11OS1961/2011|2011-07-28|A/H5N2|PB1
A/mallard/Ohio/11OS1966/2011|2011-08-01|A/H10N3|PB1
A/mallard/Mississippi/12OS443/2012|2012-01-18|A/H10N1|PB1
A/white-winged scoter/Wisconsin/10OS3922/2010|2010-11-16|A/H14N8|PB1
A/mallard/Maryland/13OS3036/2014|2014-01-11|A/H1N1|PB1
A/mallard/California/3134/2010|2010-11-24|A/H1N1|PB1
A/blue-winged teal/Canada/3237/2011|2011-09-19|A/H4N6|PB1
A/mallard/Maryland/13OS2946/2013|2013-11-23|A/H5N2|PB1
A/mallard/California/2532V/2011|2011-08-12|A/H4N6|PB1
A/mallard/Interior Alaska/10BM11421R0/2010|2010-08-12|A/H3N8|PB1
A/mallard/Illinois/10OS3676/2010|2010-10-31|A/H3N8|PB1
A/American green-winged teal/Wisconsin/10OS2847/2010|2010-10-02|A/H1N1|PB1
A/mallard/California/1154/2010|2010-07-27|A/H4N6|PB1
A/American green-winged teal/Mississippi/300/2010|2010-01-16|A/H11N9|PB1
A/mallard/Wisconsin/11OS4115/2011|2011-10-16|A/H11N9|PB1
A/northern pintail/California/2548/2010|2010-10-23|A/H11N2|PB1
A/environment/California/7451/2010|2010-02-28|A/H7N3|PB1
A/blue-winged teal/Guatemala/CIP049-04/2010|2010-01-31|A/H8N4|PB1
A/mallard/Illinois/10OS4078/2010|2010-11-28|A/H10N7|PB1
A/northern shoveler/Missouri/10OS4673/2010|2010-12-12|A/H14N6|PB1
A/mallard/Missouri/10MO0551/2010|2010-12-29|A/H7N7|PB1
A/blue-winged teal/Illinois/10OS2988/2010|2010-09-19|A/H4N6|PB1
A/common teal/Mongolia/2299/2011|2011-10-10|A/H4N6|PB1
A/chicken/Taiwan/1680/2013|2013-01-29|A/H5N2|PB1
A/chicken/Taiwan/683/2012|2012-12-06|A/H5N2|PB1
A/chicken/Taiwan/TC147/2010|2010-12-14|A/H6N1|PB1
A/chicken/Taiwan/A1997/2012|2012-02-15|A/H5N2|PB1
A/duck/Hokkaido/95/2014|2014-09-23|A/H8N4|PB1
A/common teal/Mongolia/1906/2011|2011-09-18|A/H3N8|PB1
A/wild duck/Korea/CSM42-34/2011|2011-03|A/H7N9|PB1
A/duck/Taiwan/755/2013|2013-01-10|A/H5N2|PB1
A/chicken/India/01CA06/2012|2012-01-04|A/H5N1|PB1
A/Environment/Zhenjiang/zj35/2014|2014-04-03|A/H5N8|PB1
A/duck/Jiangsu/1-15/2011|2011-01-12|A/H4N2|PB1
A/common teal/Hong Kong/MPM1740/2011|2011|A/H7N7|PB1
A/duck/Jiangsu/m234/2012|2012-01-18|A/H5N2|PB1
A/migratory duck/Jiangxi/33038/2013|2013-12-15|A/H10N7|PB1
A/duck/Hunan/S4120/2011|2011-11-11|A/H5N2|PB1
A/common Teal/Republic of Georgia/1/2011|2011-09-19|A/H3N8|PB1
A/duck/Hunan/S11547/2012|2012-03-24|A/H4N9|PB1
A/turkey/France/10-040/2010|2010-04|A/H6N1|PB1
A/duck/Jiangsu/4/2010|2010-03|A/H3N6|PB1
A/duck/Jiangxi/21714/2011|2011|A/H11N9|PB1
A/duck/Jiangsu/10-d4/2011|2011-10|A/H11N3|PB1
A/duck/Guangxi/GXd-7/2011|2011-01-14|A/H6N6|PB1
A/duck/Hunan/S1661/2012|2012-03-24|A/H6N6|PB1
A/duck/Hubei/10/2010|2010-05-22|A/H6N6|PB1
A/sparrow/Shanghai/09/2013|2013-05-05|A/H9N2|PB1
A/duck/Hunan/S4443/2011|2011-11-13|A/H11N9|PB1
A/duck/Hunan/S11205/2012|2012-03-25|A/H10N3|PB1
A/duck/Guangdong/W12/2011|2011-12-22|A/H3N2|PB1
A/environment/Hunan/S4304/2011|2011-11-13|A/H3N2|PB1
A/environment/Guangdong/C13280011/2013|2013-04-17|A/H9N2|PB1
A/tree sparrow/Shanghai/01/2013|2013-05-09|A/H7N9|PB1
A/duck/Guangxi/GXd-5/2010|2010-05-08|A/H6N1|PB1
A/duck/Lao/987/2010|2010-03|A/H5N1|PB1
A/chicken/Bhutan/248015/2010|2010-02-19|A/H5N1|PB1
A/wild duck/Shandong/1/2011|2011-04-12|A/H5N1|PB1
A/duck/Vietnam/NCVD-472/2010|2010-03-29|A/H5N1|PB1
A/Anas crecca/Hubei/Chenhu1623-5/2014|2014-05-06|A/H5N6|PB1
A/turtledove/Wuhan/WHBJ12/2014|2014-12|A/H5N6|PB1
A/wild duck/Shandong/2/2011|2011-04-13|A/H5N1|PB1
A/whooper swan/Hokkaido/4/2011|2011-01|A/H5N1|PB1
NAm
EA
0.02

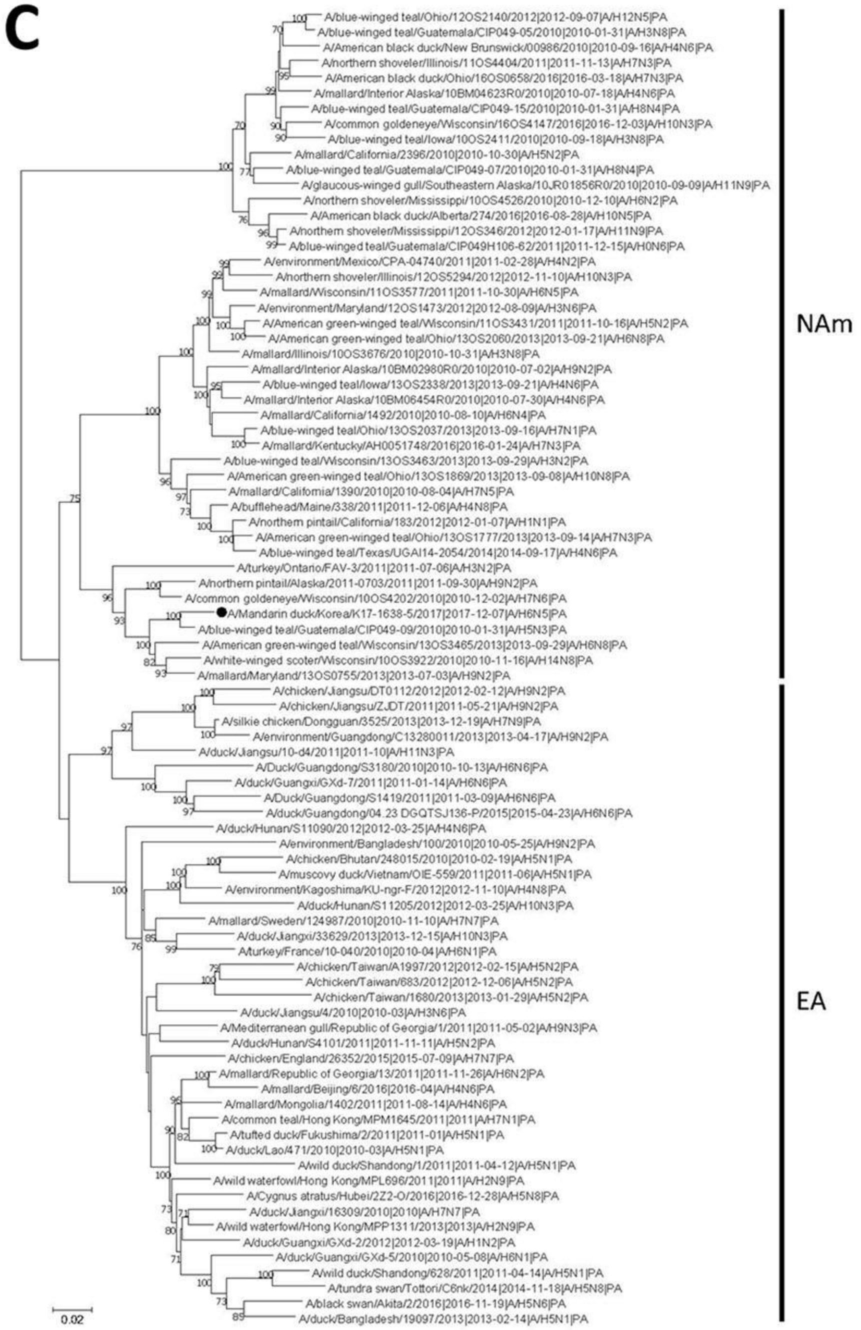
C
A/blue-winged teal/Ohio/12OS2140/2012|2012-09-07|A/H12N5|PA
A/blue-winged teal/Guatemala/CIP049-05/2010|2010-01-31|A/H3N8|PA
A/American black duck/New Brunswick/00986/2010|2010-09-16|A/H4N6|PA
A/northern shoveler/Illinois/11OS4404/2011|2011-11-13|A/H7N3|PA
A/American black duck/Ohio/16OS0658/2016|2016-03-18|A/H7N3|PA
A/mallard/Interior Alaska/10BM04623R0/2010|2010-07-18|A/H4N6|PA
A/blue-winged teal/Guatemala/CIP049-15/2010|2010-01-31|A/H8N4|PA
A/common goldeneye/Wisconsin/16OS4147/2016|2016-12-03|A/H10N3|PA
A/blue-winged teal/Iowa/10OS2411/2010|2010-09-18|A/H3N8|PA
A/mallard/California/2396/2010|2010-10-30|A/H5N2|PA
A/blue-winged teal/Guatemala/CIP049-07/2010|2010-01-31|A/H8N4|PA
A/glaucous-winged gull/Southeastern Alaska/10JR01856R0/2010|2010-09-09|A/H11N9|PA
A/northern shoveler/Mississippi/10OS4526/2010|2010-12-10|A/H6N2|PA
A/American black duck/Alberta/274/2016|2016-08-28|A/H10N5|PA
A/northern shoveler/Mississippi/12OS346/2012|2012-01-17|A/H11N9|PA
A/blue-winged teal/Guatemala/CIP049H106-62/2011|2011-12-15|A/H0N6|PA
A/environment/Mexico/CPA-04740/2011|2011-02-28|A/H4N2|PA
A/northern shoveler/Illinois/12OS5294/2012|2012-11-10|A/H10N3|PA
A/mallard/Wisconsin/11OS3577/2011|2011-10-30|A/H6N5|PA
A/environment/Maryland/12OS1473/2012|2012-08-09|A/H3N6|PA
A/American green-winged teal/Wisconsin/11OS3431/2011|2011-10-16|A/H5N2|PA
A/American green-winged teal/Ohio/13OS2060/2013|2013-09-21|A/H6N8|PA
A/mallard/Illinois/10OS3676/2010|2010-10-31|A/H3N8|PA
A/mallard/Interior Alaska/10BM02980R0/2010|2010-07-02|A/H9N2|PA
A/blue-winged teal/Iowa/13OS2338/2013|2013-09-21|A/H4N6|PA
A/mallard/Interior Alaska/10BM06454R0/2010|2010-07-30|A/H4N6|PA
A/mallard/California/1492/2010|2010-08-10|A/H6N4|PA
A/blue-winged teal/Ohio/13OS2037/2013|2013-09-16|A/H7N1|PA
A/mallard/Kentucky/AH0051748/2016|2016-01-24|A/H7N3|PA
A/blue-winged teal/Wisconsin/13OS3463/2013|2013-09-29|A/H3N2|PA
A/American green-winged teal/Ohio/13OS1869/2013|2013-09-08|A/H10N8|PA
A/mallard/California/1390/2010|2010-08-04|A/H7N5|PA
A/bufflehead/Maine/338/2011|2011-12-06|A/H4N8|PA
A/northern pintail/California/183/2012|2012-01-07|A/H1N1|PA
A/American green-winged teal/Ohio/13OS1777/2013|2013-09-14|A/H7N3|PA
A/blue-winged teal/Texas/UGAI14-2054/2014|2014-09-17|A/H4N6|PA
A/turkey/Ontario/FAV-3/2011|2011-07-06|A/H3N2|PA
A/northern pintail/Alaska/2011-0703/2011|2011-09-30|A/H9N2|PA
A/common goldeneye/Wisconsin/10OS4202/2010|2010-12-02|A/H7N6|PA
A/Mandarin duck/Korea/K17-1638-5/2017|2017-12-07|A/H6N5|PA
A/blue-winged teal/Guatemala/CIP049-09/2010|2010-01-31|A/H5N3|PA
A/American green-winged teal/Wisconsin/13OS3465/2013|2013-09-29|A/H6N8|PA
A/white-winged scoter/Wisconsin/10OS3922/2010|2010-11-16|A/H14N8|PA
A/mallard/Maryland/13OS0755/2013|2013-07-03|A/H9N2|PA
A/chicken/Jiangsu/DT0112/2012|2012-02-12|A/H9N2|PA
A/chicken/Jiangsu/ZJDT/2011|2011-05-21|A/H9N2|PA
A/silkie chicken/Dongguan/3525/2013|2013-12-19|A/H7N9|PA
A/environment/Guangdong/C13280011/2013|2013-04-17|A/H9N2|PA
A/duck/Jiangsu/10-d4/2011|2011-10|A/H11N3|PA
A/Duck/Guangdong/S3180/2010|2010-10-13|A/H6N6|PA
A/duck/Guangxi/GXd-7/2011|2011-01-14|A/H6N6|PA
A/Duck/Guangdong/S1419/2011|2011-03-09|A/H6N6|PA
A/duck/Guangdong/04.23 DGQTSJ136-P/2015|2015-04-23|A/H6N6|PA
A/duck/Hunan/S11090/2012|2012-03-25|A/H4N6|PA
A/environment/Bangladesh/100/2010|2010-05-25|A/H9N2|PA
A/chicken/Bhutan/248015/2010|2010-02-19|A/H5N1|PA
A/muscovy duck/Vietnam/OIE-559/2011|2011-06|A/H5N1|PA
A/environment/Kagoshima/KU-ngr-F/2012|2012-11-10|A/H4N8|PA
A/duck/Hunan/S11205/2012|2012-03-25|A/H10N3|PA
A/mallard/Sweden/124987/2010|2010-11-10|A/H7N7|PA
A/duck/Jiangxi/33629/2013|2013-12-15|A/H10N3|PA
A/turkey/France/10-040/2010|2010-04|A/H6N1|PA
A/chicken/Taiwan/A1997/2012|2012-02-15|A/H5N2|PA
A/chicken/Taiwan/683/2012|2012-12-06|A/H5N2|PA
A/chicken/Taiwan/1680/2013|2013-01-29|A/H5N2|PA
A/duck/Jiangsu/4/2010|2010-03|A/H3N6|PA
A/Mediterranean gull/Republic of Georgia/1/2011|2011-05-02|A/H9N3|PA
A/duck/Hunan/S4101/2011|2011-11-11|A/H5N2|PA
A/chicken/England/26352/2015|2015-07-09|A/H7N7|PA
A/mallard/Republic of Georgia/13/2011|2011-11-26|A/H6N2|PA
A/mallard/Beijing/6/2016|2016-04|A/H4N6|PA
A/mallard/Mongolia/1402/2011|2011-08-14|A/H4N6|PA
A/common teal/Hong Kong/MPM1645/2011|2011|A/H7N1|PA
A/tufted duck/Fukushima/2/2011|2011-01|A/H5N1|PA
A/duck/Lao/471/2010|2010-03|A/H5N1|PA
A/wild duck/Shandong/1/2011|2011-04-12|A/H5N1|PA
A/wild waterfowl/Hong Kong/MPL696/2011|2011|A/H2N9|PA
A/Cygnus atratus/Hubei/2Z2-O/2016|2016-12-28|A/H5N8|PA
A/duck/Jiangxi/16309/2010|2010|A/H7N7|PA
A/wild waterfowl/Hong Kong/MPP1311/2013|2013|A/H2N9|PA
A/duck/Guangxi/GXd-2/2012|2012-03-19|A/H1N2|PA
A/duck/Guangxi/GXd-5/2010|2010-05-08|A/H6N1|PA
A/wild duck/Shandong/628/2011|2011-04-14|A/H5N1|PA
A/tundra swan/Tottori/C6nk/2014|2014-11-18|A/H5N8|PA
A/black swan/Akita/2/2016|2016-11-19|A/H5N6|PA
A/duck/Bangladesh/19097/2013|2013-02-14|A/H5N1|PA
NAm
EA
0.02

D

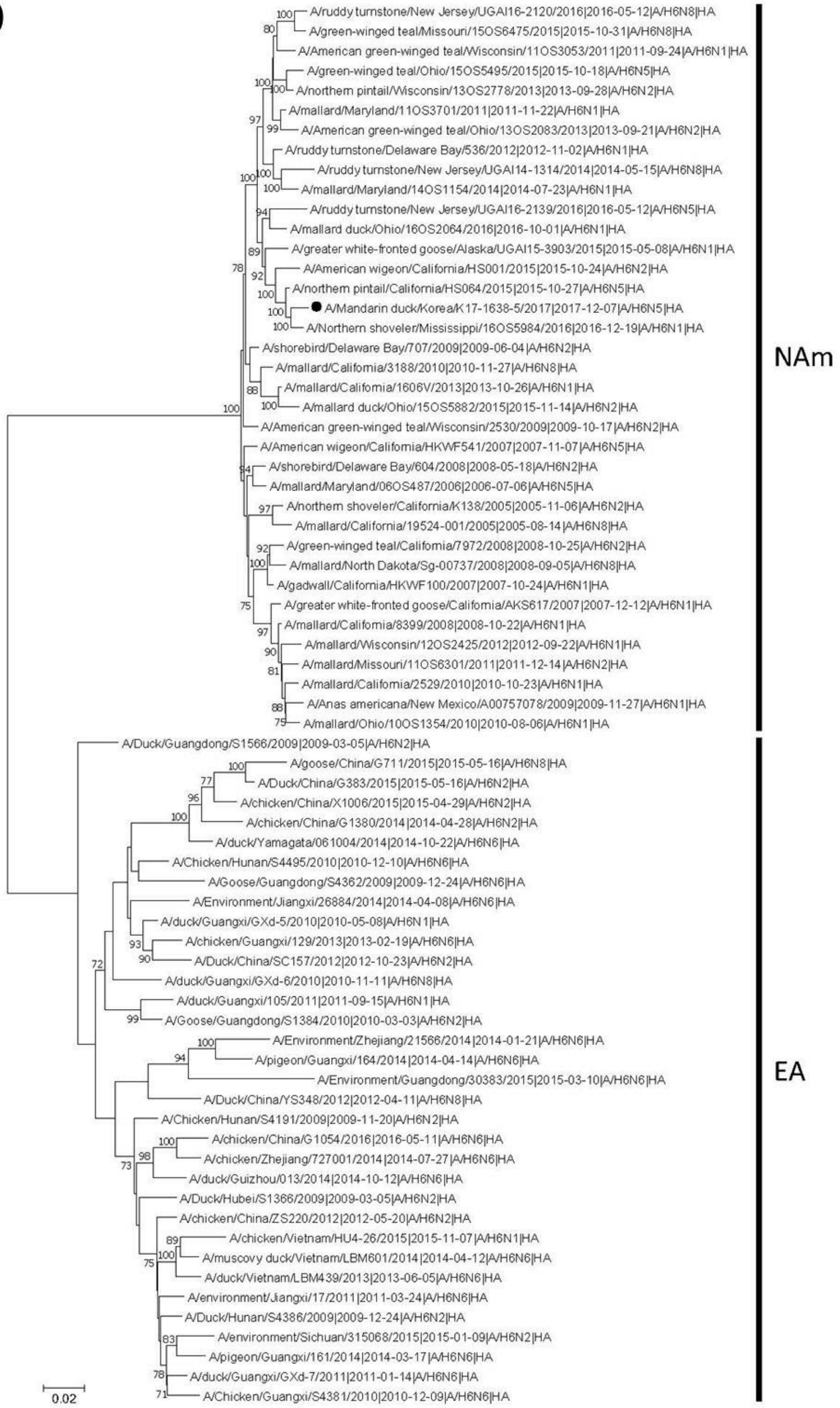
A/ruddy turnstone/New Jersey/UGAI16-2120/2016|2016-05-12|A/H6N8|HA
A/green-winged teal/Missouri/15OS6475/2015|2015-10-31|A/H6N8|HA
A/American green-winged teal/Wisconsin/11OS3053/2011|2011-09-24|A/H6N1|HA
A/green-winged teal/Ohio/15OS5495/2015|2015-10-18|A/H6N5|HA
A/northern pintail/Wisconsin/13OS2778/2013|2013-09-28|A/H6N2|HA
A/mallard/Maryland/11OS3701/2011|2011-11-22|A/H6N1|HA
A/American green-winged teal/Ohio/13OS2083/2013|2013-09-21|A/H6N2|HA
A/ruddy turnstone/Delaware Bay/536/2012|2012-11-02|A/H6N1|HA
A/ruddy turnstone/New Jersey/UGAI14-1314/2014|2014-05-15|A/H6N8|HA
A/mallard/Maryland/14OS1154/2014|2014-07-23|A/H6N1|HA
A/ruddy turnstone/New Jersey/UGAI16-2139/2016|2016-05-12|A/H6N5|HA
A/mallard duck/Ohio/16OS2064/2016|2016-10-01|A/H6N1|HA
A/greater white-fronted goose/Alaska/UGAI15-3903/2015|2015-05-08|A/H6N1|HA
A/American wigeon/California/HS001/2015|2015-10-24|A/H6N2|HA
A/northern pintail/California/HS064/2015|2015-10-27|A/H6N5|HA
A/Mandarin duck/Korea/K17-1638-5/2017|2017-12-07|A/H6N5|HA
A/Northern shoveler/Mississippi/16OS5984/2016|2016-12-19|A/H6N1|HA
A/shorebird/Delaware Bay/707/2009|2009-06-04|A/H6N2|HA
A/mallard/California/3188/2010|2010-11-27|A/H6N8|HA
A/mallard/California/1606V/2013|2013-10-26|A/H6N1|HA
A/mallard duck/Ohio/15OS5882/2015|2015-11-14|A/H6N2|HA
A/American green-winged teal/Wisconsin/2530/2009|2009-10-17|A/H6N2|HA
A/American wigeon/California/HKWF541/2007|2007-11-07|A/H6N5|HA
A/shorebird/Delaware Bay/604/2008|2008-05-18|A/H6N2|HA
A/mallard/Maryland/06OS487/2006|2006-07-06|A/H6N5|HA
A/northern shoveler/California/K138/2005|2005-11-06|A/H6N2|HA
A/mallard/California/19524-001/2005|2005-08-14|A/H6N8|HA
A/green-winged teal/California/7972/2008|2008-10-25|A/H6N2|HA
A/mallard/North Dakota/Sg-00737/2008|2008-09-05|A/H6N8|HA
A/gadwall/California/HKWF100/2007|2007-10-24|A/H6N1|HA
A/greater white-fronted goose/California/AKS617/2007|2007-12-12|A/H6N1|HA
A/mallard/California/8399/2008|2008-10-22|A/H6N1|HA
A/mallard/Wisconsin/12OS2425/2012|2012-09-22|A/H6N1|HA
A/mallard/Missouri/11OS6301/2011|2011-12-14|A/H6N2|HA
A/mallard/California/2529/2010|2010-10-23|A/H6N1|HA
A/Anas americana/New Mexico/A00757078/2009|2009-11-27|A/H6N1|HA
A/mallard/Ohio/10OS1354/2010|2010-08-06|A/H6N1|HA
A/Duck/Guangdong/S1566/2009|2009-03-05|A/H6N2|HA
A/goose/China/G711/2015|2015-05-16|A/H6N8|HA
A/Duck/China/G383/2015|2015-05-16|A/H6N2|HA
A/chicken/China/X1006/2015|2015-04-29|A/H6N2|HA
A/chicken/China/G1380/2014|2014-04-28|A/H6N2|HA
A/duck/Yamagata/061004/2014|2014-10-22|A/H6N6|HA
A/Chicken/Hunan/S4495/2010|2010-12-10|A/H6N6|HA
A/Goose/Guangdong/S4362/2009|2009-12-24|A/H6N6|HA
A/Environment/Jiangxi/26884/2014|2014-04-08|A/H6N6|HA
A/duck/Guangxi/GXd-5/2010|2010-05-08|A/H6N1|HA
A/chicken/Guangxi/129/2013|2013-02-19|A/H6N6|HA
A/Duck/China/SC157/2012|2012-10-23|A/H6N2|HA
A/duck/Guangxi/GXd-6/2010|2010-11-11|A/H6N8|HA
A/duck/Guangxi/105/2011|2011-09-15|A/H6N1|HA
A/Goose/Guangdong/S1384/2010|2010-03-03|A/H6N2|HA
A/Environment/Zhejiang/21566/2014|2014-01-21|A/H6N6|HA
A/pigeon/Guangxi/164/2014|2014-04-14|A/H6N6|HA
A/Environment/Guangdong/30383/2015|2015-03-10|A/H6N6|HA
A/Duck/China/YS348/2012|2012-04-11|A/H6N8|HA
A/Chicken/Hunan/S4191/2009|2009-11-20|A/H6N2|HA
A/chicken/China/G1054/2016|2016-05-11|A/H6N6|HA
A/chicken/Zhejiang/727001/2014|2014-07-27|A/H6N6|HA
A/duck/Guizhou/013/2014|2014-10-12|A/H6N6|HA
A/Duck/Hubei/S1366/2009|2009-03-05|A/H6N2|HA
A/chicken/China/ZS220/2012|2012-05-20|A/H6N2|HA
A/chicken/Vietnam/HU4-26/2015|2015-11-07|A/H6N1|HA
A/muscovy duck/Vietnam/LBM601/2014|2014-04-12|A/H6N6|HA
A/duck/Vietnam/LBM439/2013|2013-06-05|A/H6N6|HA
A/environment/Jiangxi/17/2011|2011-03-24|A/H6N6|HA
A/Duck/Hunan/S4386/2009|2009-12-24|A/H6N2|HA
A/environment/Sichuan/315068/2015|2015-01-09|A/H6N2|HA
A/pigeon/Guangxi/161/2014|2014-03-17|A/H6N6|HA
A/duck/Guangxi/GXd-7/2011|2011-01-14|A/H6N6|HA
A/Chicken/Guangxi/S4381/2010|2010-12-09|A/H6N6|HA
NAm
EA
0.02

E

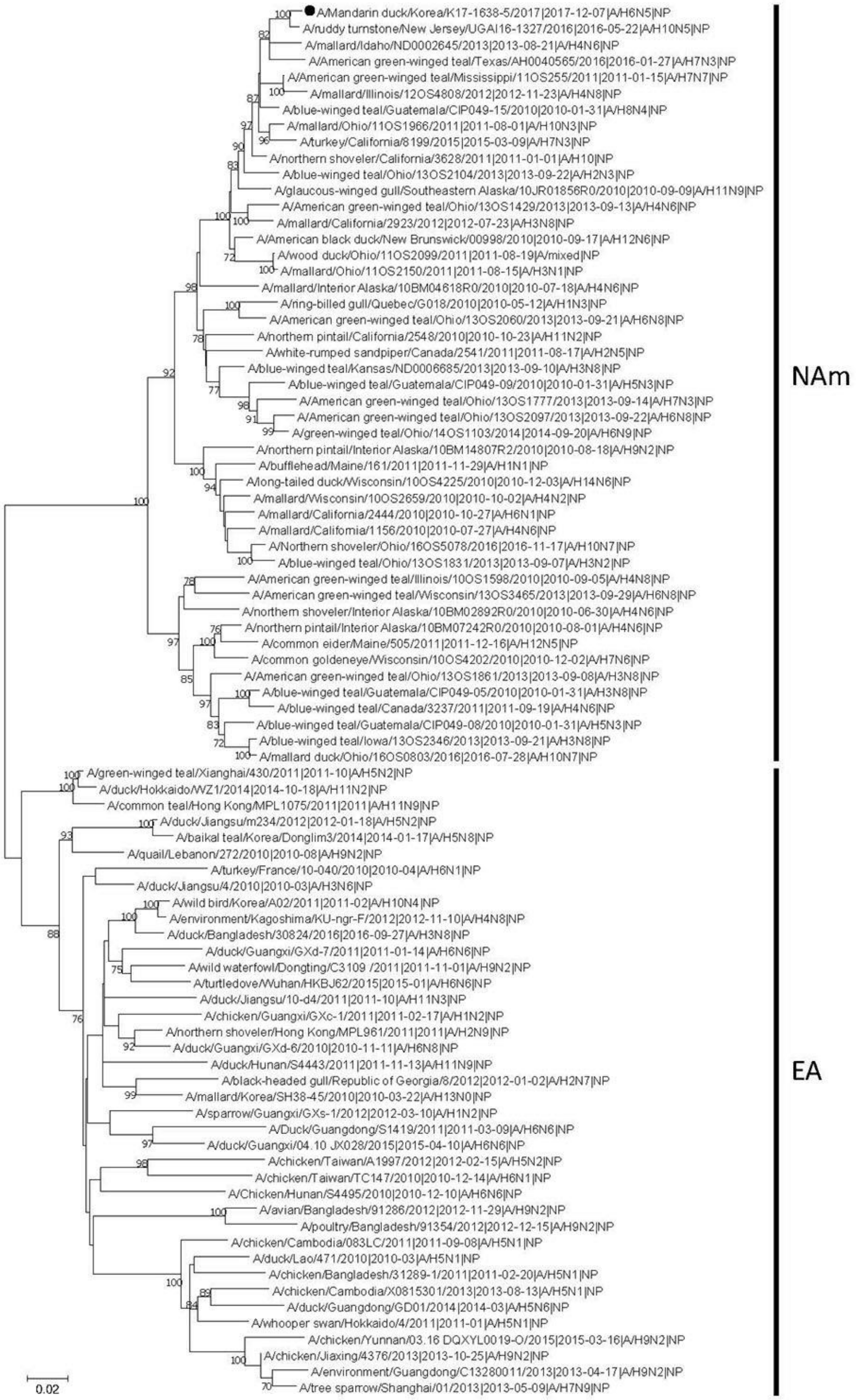

A/Mandarin duck/Korea/K17-1638-5/2017|2017-12-07|A/H6N5|NP
A/ruddy turnstone/New Jersey/UGAI16-1327/2016|2016-05-22|A/H10N5|NP
A/mallard/Idaho/ND0002645/2013|2013-08-21|A/H4N6|NP
A/American green-winged teal/Texas/AH0040565/2016|2016-01-27|A/H7N3|NP
A/American green-winged teal/Mississippi/11OS255/2011|2011-01-15|A/H7N7|NP
A/mallard/Illinois/12OS4808/2012|2012-11-23|A/H4N8|NP
A/blue-winged teal/Guatemala/CIP049-15/2010|2010-01-31|A/H8N4|NP
A/mallard/Ohio/11OS1966/2011|2011-08-01|A/H10N3|NP
A/turkey/California/8199/2015|2015-03-09|A/H7N3|NP
A/northern shoveler/California/3628/2011|2011-01-01|A/H10|NP
A/blue-winged teal/Ohio/13OS2104/2013|2013-09-22|A/H2N3|NP
A/glaucous-winged gull/Southeastern Alaska/10JR01856R0/2010|2010-09-09|A/H11N9|NP
A/American green-winged teal/Ohio/13OS1429/2013|2013-09-13|A/H4N6|NP
A/mallard/California/2923/2012|2012-07-23|A/H3N8|NP
A/American black duck/New Brunswick/00998/2010|2010-09-17|A/H12N6|NP
A/wood duck/Ohio/11OS2099/2011|2011-08-19|A/mixed|NP
A/mallard/Ohio/11OS2150/2011|2011-08-15|A/H3N1|NP
A/mallard/Interior Alaska/10BM04618R0/2010|2010-07-18|A/H4N6|NP
A/ring-billed gull/Quebec/G018/2010|2010-05-12|A/H1N3|NP
A/American green-winged teal/Ohio/13OS2060/2013|2013-09-21|A/H6N8|NP
A/northern pintail/California/2548/2010|2010-10-23|A/H11N2|NP
A/white-rumped sandpiper/Canada/2541/2011|2011-08-17|A/H2N5|NP
A/blue-winged teal/Kansas/ND0006685/2013|2013-09-10|A/H3N8|NP
A/blue-winged teal/Guatemala/CIP049-09/2010|2010-01-31|A/H5N3|NP
A/American green-winged teal/Ohio/13OS1777/2013|2013-09-14|A/H7N3|NP
A/American green-winged teal/Ohio/13OS2097/2013|2013-09-22|A/H6N8|NP
A/green-winged teal/Ohio/14OS1103/2014|2014-09-20|A/H6N9|NP
A/northern pintail/Interior Alaska/10BM14807R2/2010|2010-08-18|A/H9N2|NP
A/bufflehead/Maine/161/2011|2011-11-29|A/H1N1|NP
A/long-tailed duck/Wisconsin/10OS4225/2010|2010-12-03|A/H14N6|NP
A/mallard/Wisconsin/10OS2659/2010|2010-10-02|A/H4N2|NP
A/mallard/California/2444/2010|2010-10-27|A/H6N1|NP
A/mallard/California/1156/2010|2010-07-27|A/H4N6|NP
A/Northern shoveler/Ohio/16OS5078/2016|2016-11-17|A/H10N7|NP
A/blue-winged teal/Ohio/13OS1831/2013|2013-09-07|A/H3N2|NP
A/American green-winged teal/Illinois/10OS1598/2010|2010-09-05|A/H4N8|NP
A/American green-winged teal/Wisconsin/13OS3465/2013|2013-09-29|A/H6N8|NP
A/northern shoveler/Interior Alaska/10BM02892R0/2010|2010-06-30|A/H4N6|NP
A/northern pintail/Interior Alaska/10BM07242R0/2010|2010-08-01|A/H4N6|NP
A/common eider/Maine/505/2011|2011-12-16|A/H12N5|NP
A/common goldeneye/Wisconsin/10OS4202/2010|2010-12-02|A/H7N6|NP
A/American green-winged teal/Ohio/13OS1861/2013|2013-09-08|A/H3N8|NP
A/blue-winged teal/Guatemala/CIP049-05/2010|2010-01-31|A/H3N8|NP
A/blue-winged teal/Canada/3237/2011|2011-09-19|A/H4N6|NP
A/blue-winged teal/Guatemala/CIP049-08/2010|2010-01-31|A/H5N3|NP
A/blue-winged teal/Iowa/13OS2346/2013|2013-09-21|A/H3N8|NP
A/mallard duck/Ohio/16OS0803/2016|2016-07-28|A/H10N7|NP
A/green-winged teal/Xianghai/430/2011|2011-10|A/H5N2|NP
A/duck/Hokkaido/WZ1/2014|2014-10-18|A/H11N2|NP
A/common teal/Hong Kong/MPL1075/2011|2011|A/H11N9|NP
A/duck/Jiangsu/m234/2012|2012-01-18|A/H5N2|NP
A/baikal teal/Korea/Donglim3/2014|2014-01-17|A/H5N8|NP
A/quail/Lebanon/272/2010|2010-08|A/H9N2|NP
A/turkey/France/10-040/2010|2010-04|A/H6N1|NP
A/duck/Jiangsu/4/2010|2010-03|A/H3N6|NP
A/wild bird/Korea/A02/2011|2011-02|A/H10N4|NP
A/environment/Kagoshima/KU-ngr-F/2012|2012-11-10|A/H4N8|NP
A/duck/Bangladesh/30824/2016|2016-09-27|A/H3N8|NP
A/duck/Guangxi/GXd-7/2011|2011-01-14|A/H6N6|NP
A/wild waterfowl/Dongting/C3109 /2011|2011-11-01|A/H9N2|NP
A/turtledove/Wuhan/HKBJ62/2015|2015-01|A/H6N6|NP
A/duck/Jiangsu/10-d4/2011|2011-10|A/H11N3|NP
A/chicken/Guangxi/GXc-1/2011|2011-02-17|A/H1N2|NP
A/northern shoveler/Hong Kong/MPL961/2011|2011|A/H2N9|NP
A/duck/Guangxi/GXd-6/2010|2010-11-11|A/H6N8|NP
A/duck/Hunan/S4443/2011|2011-11-13|A/H11N9|NP
A/black-headed gull/Republic of Georgia/8/2012|2012-01-02|A/H2N7|NP
A/mallard/Korea/SH38-45/2010|2010-03-22|A/H13N0|NP
A/sparrow/Guangxi/GXs-1/2012|2012-03-10|A/H1N2|NP
A/Duck/Guangdong/S1419/2011|2011-03-09|A/H6N6|NP
A/duck/Guangxi/04.10 JX028/2015|2015-04-10|A/H6N6|NP
A/chicken/Taiwan/A1997/2012|2012-02-15|A/H5N2|NP
A/chicken/Taiwan/TC147/2010|2010-12-14|A/H6N1|NP
A/Chicken/Hunan/S4495/2010|2010-12-10|A/H6N6|NP
A/avian/Bangladesh/91286/2012|2012-11-29|A/H9N2|NP
A/poultry/Bangladesh/91354/2012|2012-12-15|A/H9N2|NP
A/chicken/Cambodia/083LC/2011|2011-09-08|A/H5N1|NP
A/duck/Lao/471/2010|2010-03|A/H5N1|NP
A/chicken/Bangladesh/31289-1/2011|2011-02-20|A/H5N1|NP
A/chicken/Cambodia/X0815301/2013|2013-08-13|A/H5N1|NP
A/duck/Guangdong/GD01/2014|2014-03|A/H5N6|NP
A/whooper swan/Hokkaido/4/2011|2011-01|A/H5N1|NP
A/chicken/Yunnan/03.16 DQXYL0019-O/2015|2015-03-16|A/H9N2|NP
A/chicken/Jiaxing/4376/2013|2013-10-25|A/H9N2|NP
A/environment/Guangdong/C13280011/2013|2013-04-17|A/H9N2|NP
A/tree sparrow/Shanghai/01/2013|2013-05-09|A/H7N9|NP
NAm
EA
0.02

F

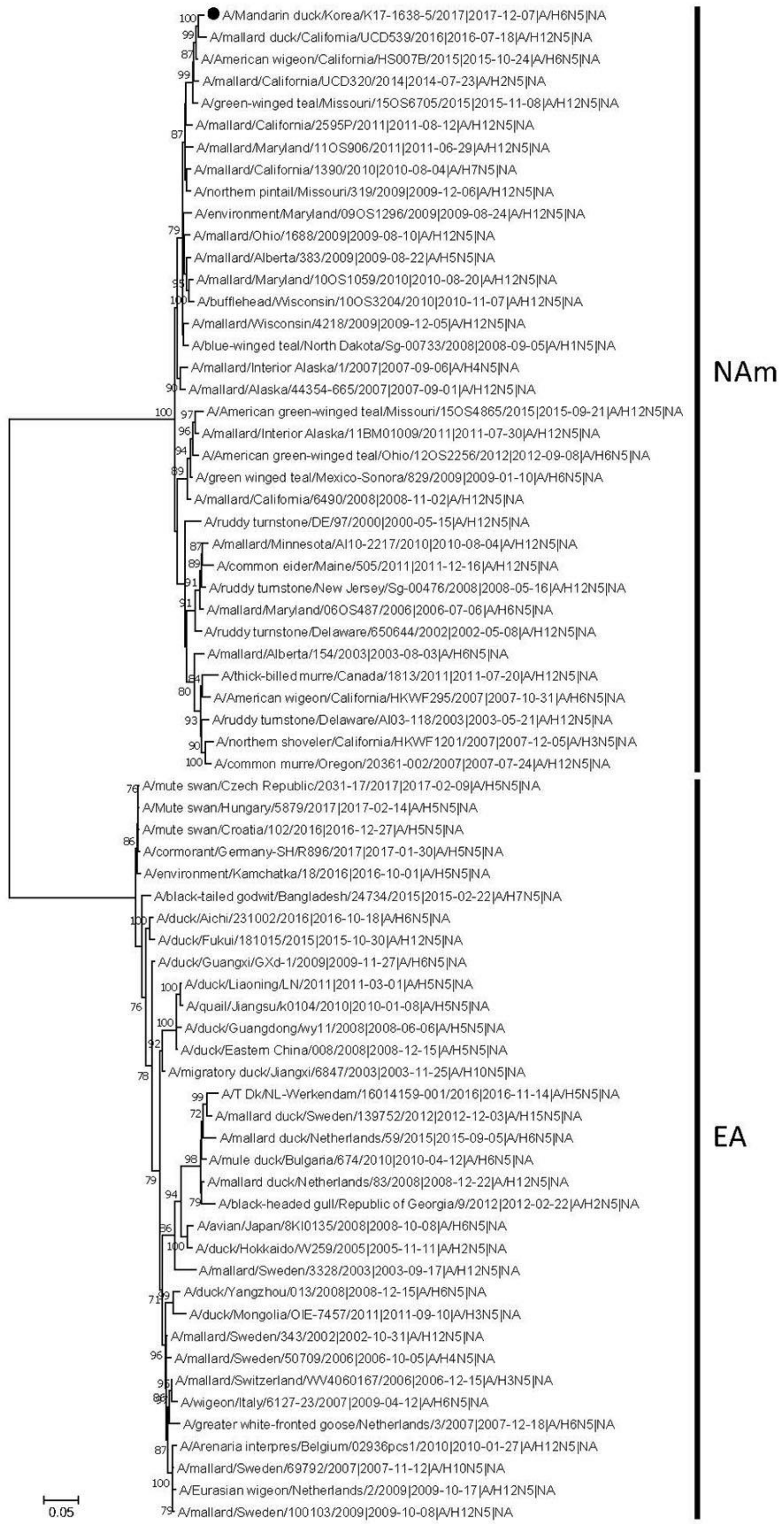
A/Mandarin duck/Korea/K17-1638-5/2017|2017-12-07|A/H6N5|NA
A/mallard duck/California/UCD539/2016|2016-07-18|A/H12N5|NA
A/American wigeon/California/HS007B/2015|2015-10-24|A/H6N5|NA
A/mallard/California/UCD320/2014|2014-07-23|A/H2N5|NA
A/green-winged teal/Missouri/15OS6705/2015|2015-11-08|A/H12N5|NA
A/mallard/California/2595P/2011|2011-08-12|A/H12N5|NA
A/mallard/Maryland/11OS906/2011|2011-06-29|A/H12N5|NA
A/mallard/California/1390/2010|2010-08-04|A/H7N5|NA
A/northern pintail/Missouri/319/2009|2009-12-06|A/H12N5|NA
A/environment/Maryland/09OS1296/2009|2009-08-24|A/H12N5|NA
A/mallard/Ohio/1688/2009|2009-08-10|A/H12N5|NA
A/mallard/Alberta/383/2009|2009-08-22|A/H5N5|NA
A/mallard/Maryland/10OS1059/2010|2010-08-20|A/H12N5|NA
A/bufflehead/Wisconsin/10OS3204/2010|2010-11-07|A/H12N5|NA
A/mallard/Wisconsin/4218/2009|2009-12-05|A/H12N5|NA
A/blue-winged teal/North Dakota/Sg-00733/2008|2008-09-05|A/H1N5|NA
A/mallard/Interior Alaska/1/2007|2007-09-06|A/H4N5|NA
A/mallard/Alaska/44354-665/2007|2007-09-01|A/H12N5|NA
A/American green-winged teal/Missouri/15OS4865/2015|2015-09-21|A/H12N5|NA
A/mallard/Interior Alaska/11BM01009/2011|2011-07-30|A/H12N5|NA
A/American green-winged teal/Ohio/12OS2256/2012|2012-09-08|A/H6N5|NA
A/green winged teal/Mexico-Sonora/829/2009|2009-01-10|A/H6N5|NA
A/mallard/California/6490/2008|2008-11-02|A/H12N5|NA
A/ruddy turnstone/DE/97/2000|2000-05-15|A/H12N5|NA
A/mallard/Minnesota/AI10-2217/2010|2010-08-04|A/H12N5|NA
A/common eider/Maine/505/2011|2011-12-16|A/H12N5|NA
A/ruddy turnstone/New Jersey/Sg-00476/2008|2008-05-16|A/H12N5|NA
A/mallard/Maryland/06OS487/2006|2006-07-06|A/H6N5|NA
A/ruddy turnstone/Delaware/650644/2002|2002-05-08|A/H12N5|NA
A/mallard/Alberta/154/2003|2003-08-03|A/H6N5|NA
A/thick-billed murre/Canada/1813/2011|2011-07-20|A/H12N5|NA
A/American wigeon/California/HKWF295/2007|2007-10-31|A/H6N5|NA
A/ruddy turnstone/Delaware/AI03-118/2003|2003-05-21|A/H12N5|NA
A/northern shoveler/California/HKWF1201/2007|2007-12-05|A/H3N5|NA
A/common murre/Oregon/20361-002/2007|2007-07-24|A/H12N5|NA
A/mute swan/Czech Republic/2031-17/2017|2017-02-09|A/H5N5|NA
A/Mute swan/Hungary/5879/2017|2017-02-14|A/H5N5|NA
A/mute swan/Croatia/102/2016|2016-12-27|A/H5N5|NA
A/cormorant/Germany-SH/R896/2017|2017-01-30|A/H5N5|NA
A/environment/Kamchatka/18/2016|2016-10-01|A/H5N5|NA
A/black-tailed godwit/Bangladesh/24734/2015|2015-02-22|A/H7N5|NA
A/duck/Aichi/231002/2016|2016-10-18|A/H6N5|NA
A/duck/Fukui/181015/2015|2015-10-30|A/H12N5|NA
A/duck/Guangxi/GXd-1/2009|2009-11-27|A/H6N5|NA
A/duck/Liaoning/LN/2011|2011-03-01|A/H5N5|NA
A/quail/Jiangsu/k0104/2010|2010-01-08|A/H5N5|NA
A/duck/Guangdong/wy11/2008|2008-06-06|A/H5N5|NA
A/duck/Eastern China/008/2008|2008-12-15|A/H5N5|NA
A/migratory duck/Jiangxi/6847/2003|2003-11-25|A/H10N5|NA
A/T Dk/NL-Werkendam/16014159-001/2016|2016-11-14|A/H5N5|NA
A/mallard duck/Sweden/139752/2012|2012-12-03|A/H15N5|NA
A/mallard duck/Netherlands/59/2015|2015-09-05|A/H6N5|NA
A/mule duck/Bulgaria/674/2010|2010-04-12|A/H6N5|NA
A/mallard duck/Netherlands/83/2008|2008-12-22|A/H12N5|NA
A/black-headed gull/Republic of Georgia/9/2012|2012-02-22|A/H2N5|NA
A/avian/Japan/8KI0135/2008|2008-10-08|A/H6N5|NA
A/duck/Hokkaido/W259/2005|2005-11-11|A/H2N5|NA
A/mallard/Sweden/3328/2003|2003-09-17|A/H12N5|NA
A/duck/Yangzhou/013/2008|2008-12-15|A/H6N5|NA
A/duck/Mongolia/OIE-7457/2011|2011-09-10|A/H3N5|NA
A/mallard/Sweden/343/2002|2002-10-31|A/H12N5|NA
A/mallard/Sweden/50709/2006|2006-10-05|A/H4N5|NA
A/mallard/Switzerland/WV4060167/2006|2006-12-15|A/H3N5|NA
A/wigeon/Italy/6127-23/2007|2009-04-12|A/H6N5|NA
A/greater white-fronted goose/Netherlands/3/2007|2007-12-18|A/H6N5|NA
A/Arenaria interpres/Belgium/02936pcs1/2010|2010-01-27|A/H12N5|NA
A/mallard/Sweden/69792/2007|2007-11-12|A/H10N5|NA
A/Eurasian wigeon/Netherlands/2/2009|2009-10-17|A/H12N5|NA
A/mallard/Sweden/100103/2009|2009-10-08|A/H12N5|NA
NAm
EA
0.05

G

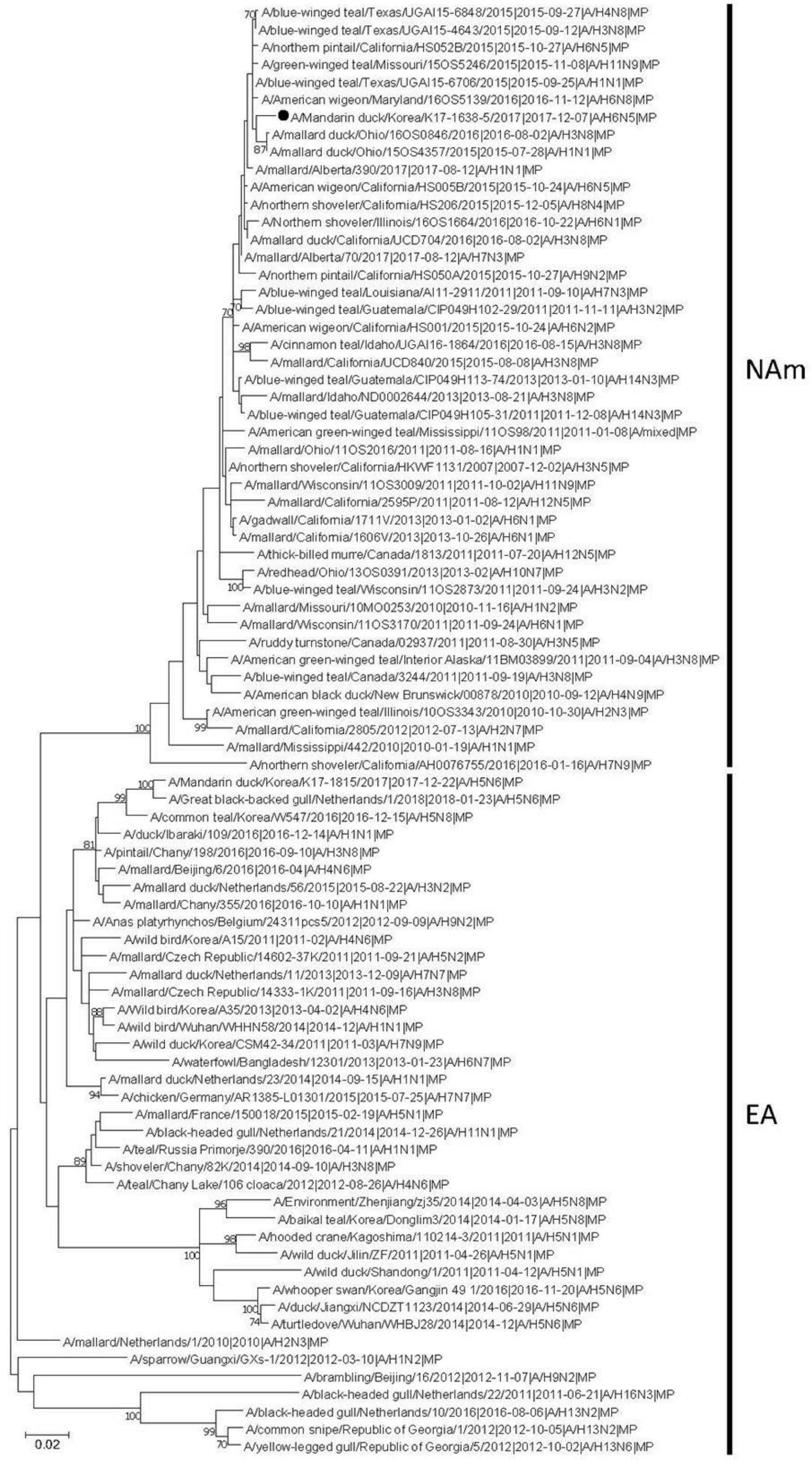
A/blue-winged teal/Texas/UGAI15-6848/2015|2015-09-27|A/H4N8|MP
A/blue-winged teal/Texas/UGAI15-4643/2015|2015-09-12|A/H3N8|MP
A/northern pintail/California/HS052B/2015|2015-10-27|A/H6N5|MP
A/green-winged teal/Missouri/15OS5246/2015|2015-11-08|A/H11N9|MP
A/blue-winged teal/Texas/UGAI15-6706/2015|2015-09-25|A/H1N1|MP
A/American wigeon/Maryland/16OS5139/2016|2016-11-12|A/H6N8|MP
A/Mandarin duck/Korea/K17-1638-5/2017|2017-12-07|A/H6N5|MP
A/mallard duck/Ohio/16OS0846/2016|2016-08-02|A/H3N8|MP
A/mallard duck/Ohio/15OS4357/2015|2015-07-28|A/H1N1|MP
A/mallard/Alberta/390/2017|2017-08-12|A/H1N1|MP
A/American wigeon/California/HS005B/2015|2015-10-24|A/H6N5|MP
A/northern shoveler/California/HS206/2015|2015-12-05|A/H8N4|MP
A/Northern shoveler/Illinois/16OS1664/2016|2016-10-22|A/H6N1|MP
A/mallard duck/California/UCD704/2016|2016-08-02|A/H3N8|MP
A/mallard/Alberta/70/2017|2017-08-12|A/H7N3|MP
A/northern pintail/California/HS050A/2015|2015-10-27|A/H9N2|MP
A/blue-winged teal/Louisiana/AI11-2911/2011|2011-09-10|A/H7N3|MP
A/blue-winged teal/Guatemala/CIP049H102-29/2011|2011-11-11|A/H3N2|MP
A/American wigeon/California/HS001/2015|2015-10-24|A/H6N2|MP
A/cinnamon teal/Idaho/UGAI16-1864/2016|2016-08-15|A/H3N8|MP
A/mallard/California/UCD840/2015|2015-08-08|A/H3N8|MP
A/blue-winged teal/Guatemala/CIP049H113-74/2013|2013-01-10|A/H14N3|MP
A/mallard/Idaho/ND0002644/2013|2013-08-21|A/H3N8|MP
A/blue-winged teal/Guatemala/CIP049H105-31/2011|2011-12-08|A/H14N3|MP
A/American green-winged teal/Mississippi/11OS98/2011|2011-01-08|A/mixed|MP
A/mallard/Ohio/11OS2016/2011|2011-08-16|A/H1N1|MP
A/northern shoveler/California/HKWF1131/2007|2007-12-02|A/H3N5|MP
A/mallard/Wisconsin/11OS3009/2011|2011-10-02|A/H11N9|MP
A/mallard/California/2595P/2011|2011-08-12|A/H12N5|MP
A/gadwall/California/1711V/2013|2013-01-02|A/H6N1|MP
A/mallard/California/1606V/2013|2013-10-26|A/H6N1|MP
A/thick-billed murre/Canada/1813/2011|2011-07-20|A/H12N5|MP
A/redhead/Ohio/13OS0391/2013|2013-02|A/H10N7|MP
A/blue-winged teal/Wisconsin/11OS2873/2011|2011-09-24|A/H3N2|MP
A/mallard/Missouri/10MO0253/2010|2010-11-16|A/H1N2|MP
A/mallard/Wisconsin/11OS3170/2011|2011-09-24|A/H6N1|MP
A/ruddy turnstone/Canada/02937/2011|2011-08-30|A/H3N5|MP
A/American green-winged teal/Interior Alaska/11BM03899/2011|2011-09-04|A/H3N8|MP
A/blue-winged teal/Canada/3244/2011|2011-09-19|A/H3N8|MP
A/American black duck/New Brunswick/00878/2010|2010-09-12|A/H4N9|MP
A/American green-winged teal/Illinois/10OS3343/2010|2010-10-30|A/H2N3|MP
A/mallard/California/2805/2012|2012-07-13|A/H2N7|MP
A/mallard/Mississippi/442/2010|2010-01-19|A/H1N1|MP
A/northern shoveler/California/AH0076755/2016|2016-01-16|A/H7N9|MP
A/Mandarin duck/Korea/K17-1815/2017|2017-12-22|A/H5N6|MP
A/Great black-backed gull/Netherlands/1/2018|2018-01-23|A/H5N6|MP
A/common teal/Korea/W547/2016|2016-12-15|A/H5N8|MP
A/duck/Ibaraki/109/2016|2016-12-14|A/H1N1|MP
A/pintail/Chany/198/2016|2016-09-10|A/H3N8|MP
A/mallard/Beijing/6/2016|2016-04|A/H4N6|MP
A/mallard duck/Netherlands/56/2015|2015-08-22|A/H3N2|MP
A/mallard/Chany/355/2016|2016-10-10|A/H1N1|MP
A/Anas platyrhynchos/Belgium/24311pcs5/2012|2012-09-09|A/H9N2|MP
A/wild bird/Korea/A15/2011|2011-02|A/H4N6|MP
A/mallard/Czech Republic/14602-37K/2011|2011-09-21|A/H5N2|MP
A/mallard duck/Netherlands/11/2013|2013-12-09|A/H7N7|MP
A/mallard/Czech Republic/14333-1K/2011|2011-09-16|A/H3N8|MP
A/Wild bird/Korea/A35/2013|2013-04-02|A/H4N6|MP
A/wild bird/Wuhan/WHHN58/2014|2014-12|A/H1N1|MP
A/wild duck/Korea/CSM42-34/2011|2011-03|A/H7N9|MP
A/waterfowl/Bangladesh/12301/2013|2013-01-23|A/H6N7|MP
A/mallard duck/Netherlands/23/2014|2014-09-15|A/H1N1|MP
A/chicken/Germany/AR1385-L01301/2015|2015-07-25|A/H7N7|MP
A/mallard/France/150018/2015|2015-02-19|A/H5N1|MP
A/black-headed gull/Netherlands/21/2014|2014-12-26|A/H11N1|MP
A/teal/Russia Primorje/390/2016|2016-04-11|A/H1N1|MP
A/shoveler/Chany/82K/2014|2014-09-10|A/H3N8|MP
A/teal/Chany Lake/106 cloaca/2012|2012-08-26|A/H4N6|MP
A/Environment/Zhenjiang/zj35/2014|2014-04-03|A/H5N8|MP
A/baikal teal/Korea/Donglim3/2014|2014-01-17|A/H5N8|MP
A/hooded crane/Kagoshima/110214-3/2011|2011|A/H5N1|MP
A/wild duck/Jilin/ZF/2011|2011-04-26|A/H5N1|MP
A/wild duck/Shandong/1/2011|2011-04-12|A/H5N1|MP
A/whooper swan/Korea/Gangjin 49 1/2016|2016-11-20|A/H5N6|MP
A/duck/Jiangxi/NCDZT1123/2014|2014-06-29|A/H5N6|MP
A/turtledove/Wuhan/WHBJ28/2014|2014-12|A/H5N6|MP
A/mallard/Netherlands/1/2010|2010|A/H2N3|MP
A/sparrow/Guangxi/GXs-1/2012|2012-03-10|A/H1N2|MP
A/brambling/Beijing/16/2012|2012-11-07|A/H9N2|MP
A/black-headed gull/Netherlands/22/2011|2011-06-21|A/H16N3|MP
A/black-headed gull/Netherlands/10/2016|2016-08-06|A/H13N2|MP
A/common snipe/Republic of Georgia/1/2012|2012-10-05|A/H13N2|MP
A/yellow-legged gull/Republic of Georgia/5/2012|2012-10-02|A/H13N6|MP
0.02
NAm
EA

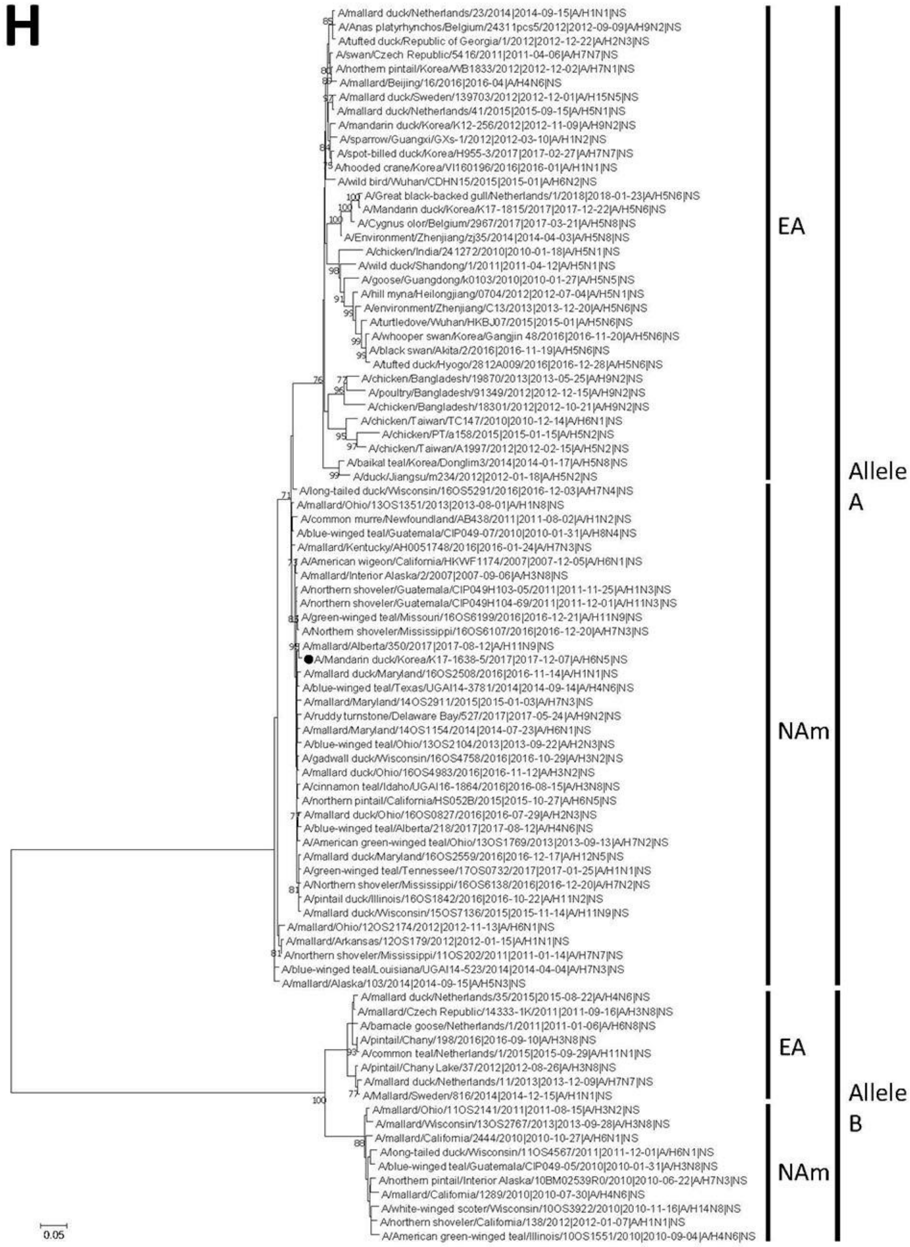

Appendix Figure 2. Maximum-likelihood phylogenetic trees for the reference strains of Eurasian (EA) and North American (NAm) lineages and A/Mandarin duck/Korea/K17–1638–5/2017(H6N5) virus (indicated with black circle). Bootstrap values >70% are shown. A) Polymerase basic 2 gene; B) polymerase basic 1 gene; C) polymerase acidic gene; D) hemagglutinin gene; E) nucleoprotein gene; F) neuraminidase gene; G) matrix gene; H) nonstructural gene.
